# Dynamics of Mycobacterium tuberculosis-Specific and Nonspecific Immune Responses in Women with Tuberculosis Infection during Pregnancy

**DOI:** 10.1128/spectrum.01178-22

**Published:** 2022-08-15

**Authors:** Fregenet Tesfaye, Erik Sturegård, John Walles, Bayissa Bekele, Kidist Bobosha, Per Björkman, Marianne Jansson

**Affiliations:** a Clinical Infection Medicine, Department of Translational Medicine, Lund University, Malmö, Sweden; b Armauer Hansen Research Institute, Addis Ababa, Ethiopia; c Department of Infectious Diseases, Central Hospital, Kristianstad, Sweden; d Adama Public Health Research and Referral Laboratory Center, Adama, Ethiopia; e Department of Infectious Diseases, Skåne University Hospital, Malmö, Sweden; f Medical Microbiology, Department of Laboratory Medicine, Lund University, Lund, Sweden; Louisiana State University Health Sciences Center

**Keywords:** tuberculosis infection, pregnancy, *Mycobacterium tuberculosis* antigen reactivity, HIV-uninfected, interleukin 2, interferon-γ inducible protein 10, transforming growth factor-beta1

## Abstract

The immune control of tuberculosis (TB) infection could be influenced by pregnancy. To elucidate this, we longitudinally characterized Mycobacterium tuberculosis (Mtb)-specific and nonspecific immune responses in women during pregnancy and postpartum. HIV-uninfected women without past or current active TB, and with blood samples available from the 1st/2nd trimester, 3rd trimester, and 9 months postpartum, were identified at Ethiopian antenatal care clinics. Twenty-two TB+ women and 10 TB− women, defined according to Mtb-stimulated interferon-γ levels (≥0.35 and <0.20 IU/mL, respectively, in the Quantiferon-TB Gold-Plus assay), were included in the study. Longitudinal dynamics of six cytokines (IL-1ra, IL-2, IP-10, MCP-2, MCP-3, and TGF-β1) were analyzed in supernatants from Mtb-stimulated and unstimulated whole blood. In TB+ women, Mtb-specific expression of IL-2 and IP-10 was higher at 3rd compared to 1st/2nd trimester (median 139 pg/mL versus 62 pg/mL, *P* = 0.006; 4,999 pg/mL versus 2,310 pg/mL, *P* = 0.031, respectively), whereas level of Mtb-triggered TGF-β1 was lower at 3rd compared to 1st/2nd trimester (−6.8 ng/mL versus 2.3 ng/mL, *P* = 0.020). Unstimulated IL-2, IP-10, and MCP-2 levels were increased postpartum, compared with those noted during pregnancy, in TB+ women. Additionally, postpartum levels of proinflammatory cytokines in unstimulated blood were higher in TB+ women, than in TB− women. None of the women developed active TB during follow-up. Taken together, dynamic changes of Mtb-specific cytokine expression revealed during the 3rd trimester in TB+ women indicate increased Mtb-antigen stimulation at later stages of pregnancy. This could reflect elevated bacterial activity, albeit without transition to active TB, during pregnancy.

**IMPORTANCE** Tuberculosis (TB) is globally one of the most common causes of death, and a quarter of the world's population is estimated to have TB infection. The risk of active TB is increased in connection to pregnancy, a phenomenon that could be due to physiological immune changes. Here, we studied the effect of pregnancy on immune responses triggered in HIV-uninfected women with TB infection, by analyzing blood samples obtained longitudinally during pregnancy and after childbirth. We found that the dynamics of Mtb-specific and nonspecific immune responses changed during pregnancy, especially in later stages of pregnancy, although none of the women followed in this study developed active TB. This suggests that incipient TB, with elevated bacterial activity, occurs during pregnancy, but progression of infection appears to be counteracted by Mtb-specific immune responses. Thus, this study sheds light on immune control of TB during pregnancy, which could be of importance for future intervention strategies.

## INTRODUCTION

The outcome of Mycobacterium tuberculosis (Mtb) infection is largely determined by the balance of different cellular immune responses that may lead to the containment of Mtb bacteria in individuals with TB infection (1, 2). Certain immune responses, especially those mediated by CD4^+^ Th1-cells, CD4^+^ Th17-cells, and cytotoxic CD8^+^ T-cells, have been linked to control of bacterial replication (3, 4), whereas regulatory T (Treg) cells and CD4^+^ Th2-cells counteract these effects (5, 6). Therefore, a predominance of Treg and Th2 responses may trigger TB progression (6, 7).

During pregnancy, the balance between these different subsets of T-cells is altered to maintain maternal tolerance to the fetus (8, 9). However, this physiological immune modification also confers increased susceptibility to various infections in pregnant women (10, 11). Registry-based studies performed in high-income countries have shown an increased risk of TB, most markedly in the postpartum period, in connection to pregnancy (12, 13). Furthermore, pregnant women with HIV living in TB-regions of endemicity have been found to be at high risk of active TB (14, 15). This could be due to pregnancy-related immune changes that perturb the control of TB infection, leading to the development of active disease. The mechanisms involved are incompletely understood and cannot be directly explored at the pulmonary site of infection in humans. However, patterns of immune mediators secreted during pregnancy and postpartum may reflect the changing status of immune control in individuals with TB infection. Longitudinal studies on interferon-γ (IFN-γ) secretion in women with HIV and TB infection performed during pregnancy and postpartum show dampening of Mtb-specific IFN-γ secretion at later stages of pregnancy (16, 17). In contrast, we have recently reported an opposite pattern in HIV-uninfected women; i.e., increased Mtb-specific responses despite lower mitogen-triggered IFN-γ secretion (18), which suggests increased Mtb-antigen stimulation at later stages of pregnancy. We hypothesize that this may indicate increased bacterial activity in granulomas of TB-infected women during pregnancy. However, this phenomenon appears to be transient, since active TB did not occur in any of these women during follow-up.

IFN-γ, a proinflammatory cytokine secreted by different immune cells, including activated Th1-cells, plays an important role in the maintenance of TB infection (19). However, the immunological balance that determines the outcome of TB infection is complex, especially during pregnancy. In this study, we aimed to further elucidate the dynamics of immune responses that occur during pregnancy in HIV-uninfected women with TB infection by analyzing cytokines secreted by different immune cells in response to Mtb-antigen stimulation. Furthermore, we assessed patterns of longitudinal nonspecific immune responses during pregnancy and postpartum in women with and without TB infection.

## RESULTS

### Participant characteristics.

Samples from 99 women were collected at three time points: 1st/2nd trimester, 3rd trimester, and postpartum. Of these, 22 HIV-uninfected TB+ women were included in the study (Fig. S1). 10 randomly selected HIV-uninfected TB− women were included as controls. The median age was 26 years. Median gestational age was 17 (IQR: 14 to 19) weeks at enrollment, and a majority of women (79%) were enrolled during the 2nd trimester. None of the women developed active TB during a median 24-months (range 9 to 48 months) follow up. Further characteristics of the study participants are presented in Table 1.

**TABLE 1:** Baseline characteristics of women included in the study

Characteristics^*a*^	Total	TB+ IFN-γ ≥0.35 IU/mL	TB− IFN-γ <0.20 IU/mL
	32	22	10
Age (yrs)^*a*^	26 (22–28)	26 (23–30)	25 (22–27)
Marital status			
Married	31(97)	21 (95)	10 (100)
Single	1 (3)	1 (5)	0 (0)
Family size			
<4 members	26 (81)	17 (77)	9 (90)
>4 members	6 (19)	5 (23)	1 (10)
Residence			
Urban	32 (100)	22 (100)	10 (100)
Permanent	24 (75)	14 (64)	10 (100)
Non-permanent	8 (25)	8 (36)	0 (0)
Single room	23 (72)	16 (73)	7 (70)
Multiple rooms	9 (28)	6 (27)	3 (30)
Education			
Illiterate	3 (9)	3 (14)	0 (0)
<6 grades	6 (19)	2 (9)	4 (40)
≥6 grades	23 (72)	17 (77)	6 (60)
Occupation			
Daily laborer	6 (19)	6 (27)	0 (0)
Permanent occupation	12 (38)	8 (36)	4 (40)
Housewife	14 (44)	8 (36)	6 (60)
MUAC^*b*,*c*^	25 (23–28)	26 (24–29)	24 (23–26)
Gestational age at enrollment (wks)^*b*,*d*^	16 (13–19)	16 (14–19)	15 (14–19)
Parity			
No previous pregnancy	10 (31)	4 (18)	6 (60)
Previous pregnancies	22 (69)	18 (82)	4 (40)

a Data are presented as number (%) unless otherwise quoted.

b Median and interquartile range in parentheses.

c MUAC: mid upper arm circumference.

d Women enrolled at the 1st versus 2nd trimester: 6 (27%) versus 16 (73%) TB+, and 3 (30%) versus 7 (70%) TB−.

### Mtb-specific cytokine responses during pregnancy and postpartum.

**FIG 1:**
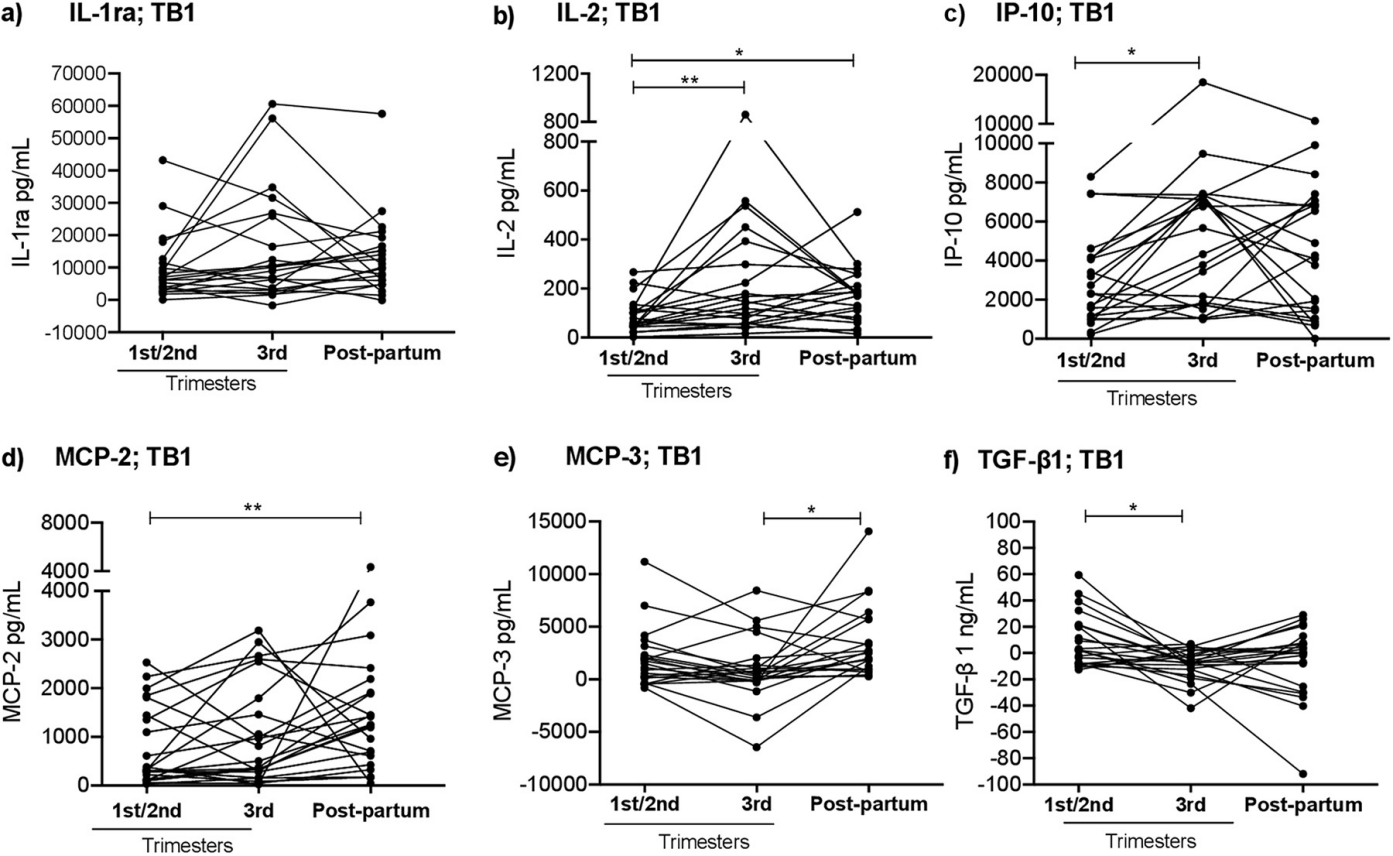
Mtb-antigen-stimulated cytokine responses in women with TB infection longitudinally sampled at the 1st/2nd and 3rd trimesters and postpartum (*n* = 22). Longitudinal analyses of QFT TB1-antigen-stimulated whole blood cytokine secretion of a) interleukin-1 receptor antagonist (IL-1ra); b) interleukin-2 (IL-2); c) IFN-γ inducible protein 10 (IP-10); d) monocyte chemoattractant protein-2 (MCP-2); e) MCP-3; and f) transforming growth factor beta 1 (TGF-β1). Statistical analyses were performed using Friedman test, followed by Dunn’s multiple-comparison test, at the longitudinal comparisons. *, *P* < 0.05 and **, *P* < 0.01. Mtb, Mycobacterium tuberculosis; QFT, QuantiFERON TB Gold Plus.

To investigate the effect of pregnancy on the dynamics of Mtb-specific immune responses in TB+ women, we compared the concentrations of IL-1ra, IL-2, IP-10, MCP-2, MCP-3, and TGF-β1 in response to QFT TB1 and TB2 stimulations at different time points during pregnancy and postpartum. TB1-triggered concentrations of IL-2 and IP-10 were significantly higher at 3rd trimester than those at 1st/2nd trimester (median IL-2: 139 pg/mL versus 62 pg/mL, *P* = 0.006; and IP-10: 4,999 pg/mL versus 2,310 pg/mL, *P* = 0.031) (Fig. 1). In contrast, TB1-triggered TGF-β1 levels were lower at 3rd trimester than those at 1st/2nd trimester (median TGF-β1: −6.8 ng/mL versus 2.3 ng/mL, *P* = 0.020) (Fig. 1). Similar dynamics were noted for the TB2-stimulated supernatants (Fig. S2). TB1-triggered IL-2 and MCP-2 levels were higher postpartum than at 1st/2nd trimester (median IL-2: 175 pg/mL versus 62 pg/mL, *P* = 0.010; MCP-2: 1,239 pg/mL versus 321 pg/mL, *P* = 0.007). Moreover, TB1-stimulated MCP-3 secretion was higher postpartum than at 3rd trimester (2,229 pg/mL versus 471 pg/mL, *P* = 0.020). TB1-stimulated levels of IL-1ra and MCP-2 also showed an increasing trend, although nonsignificant, between pregnancy and postpartum (Fig. 1). The patterns of TB2-triggered secretion of MCP-2, MCP-3, and TGF-β1 were similar to those of TB1, but median concentrations were not significantly different at different time points (Fig. S2). Median and IQR for each measured immune mediator at each time point are summarized in Table S1, and individual concentrations of TB1, nil and TB1-nil assayed mediators are given in Table S2. Furthermore, with the exception of a slight reduction in TB1-triggered MCP-3 levels at 3rd trimester, no significant differences in expression patterns of cytokines were observed in response to TB1- and TB2-antigens in TB-women (Fig. S3).

### Nonspecific cytokine responses during pregnancy and postpartum.

**FIG 2:**
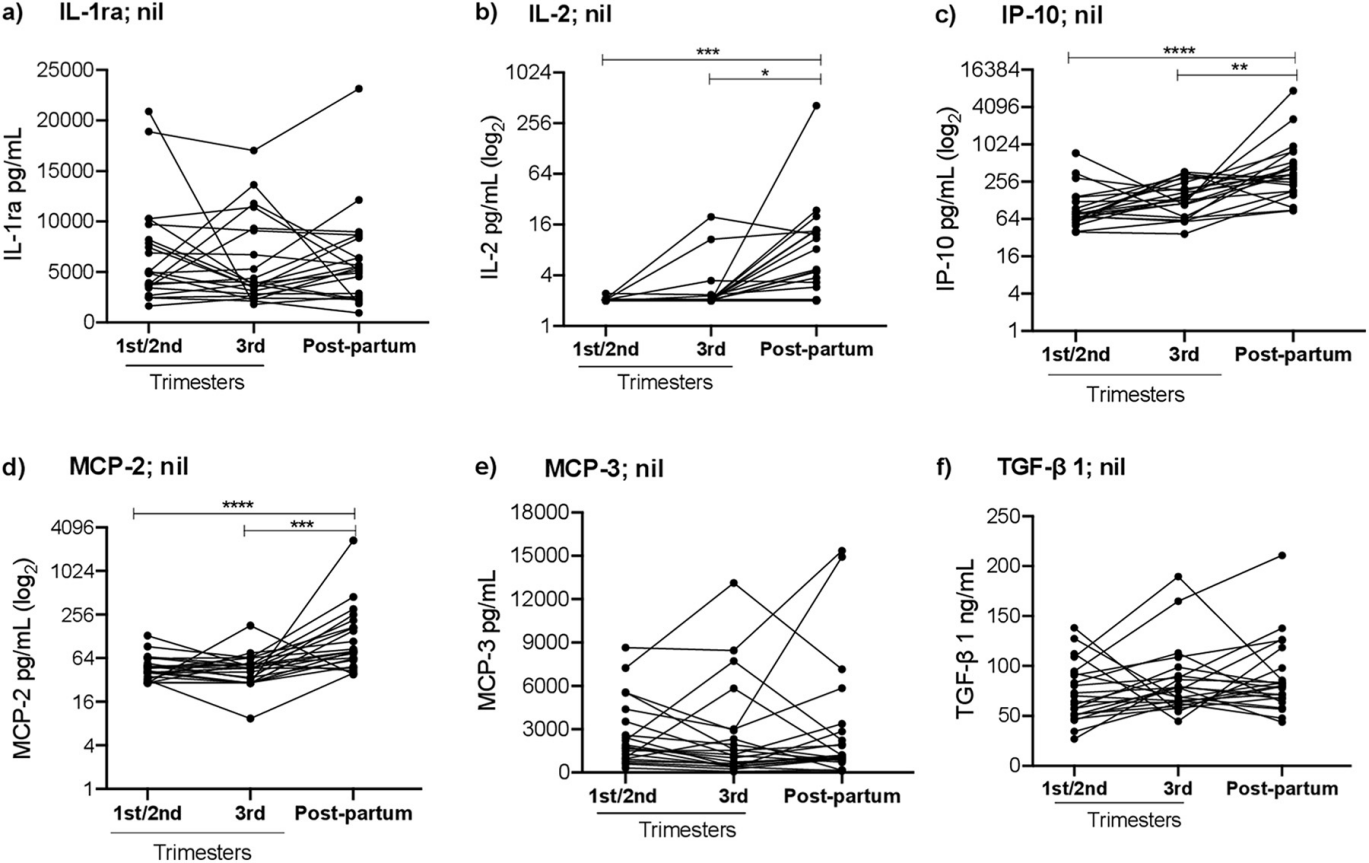
Cytokine concentrations in unstimulated whole blood QFT supernatants obtained at the 1st/2nd and 3rd trimesters and postpartum in women (*n *= 22) with TB infection. Longitudinal analyses of cytokines in unstimulated (nil) whole blood, including a) interleukin-1 receptor antagonist (IL-1ra); b) interleukin-2 (IL-2); c) IFN-γ inducible protein 10 (IP-10); d) monocyte chemoattractant protein-2 (MCP-2); e) MCP-3; and f) transforming growth factor beta 1 (TGF-β1). Statistical analyses were performed using Friedman test, followed by Dunn’s multiple-comparison test, at the longitudinal comparisons. *, *P* < 0.05; **, *P* < 0.01; ***, *P* < 0.001; and ****, *P* < 0.0001.

**FIG 3:**
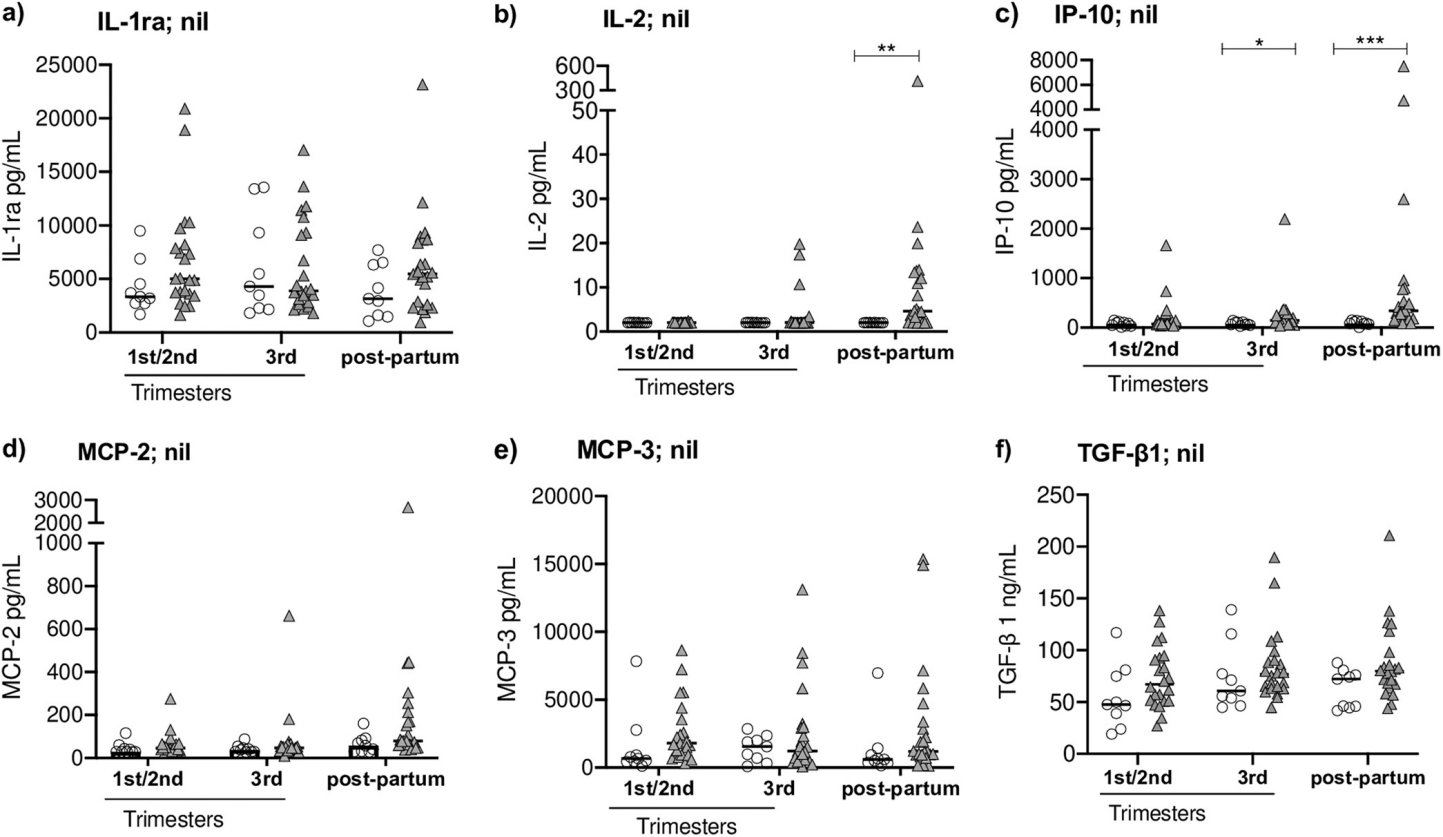
Comparisons of cytokines in unstimulated whole blood in women with and without TB infection sampled at the 1st/2nd and 3rd trimesters and postpartum. Concentrations of cytokines in QFT unstimulated (nil) whole blood QFT supernatants from women with TB infection (QFT ≥0.35 IU/mL, *n *= 22; filled triangles) and controls (QFT <0.20 IU/mL, *n *= 10; unfilled circles) including: a) interleukin-1 receptor antagonist (IL-1ra); b) interleukin-2 (IL-2); c) IFN-γ inducible protein 10 (IP-10); d) monocyte chemoattractant protein-2 (MCP-2); e) MCP-3; and f) transforming growth factor beta 1 (TGF-β1). Statistical analyses were performed using Mann-Whitney U-test, followed by Bonferroni-Dunn’s method at comparisons between groups at each time point. *, *P* < 0.05; **, *P* < 0.01; and ***, *P* < 0.001. QFT, QuantiFERON TB Gold Plus.

To study how pregnancy affects the dynamics of systemic inflammation markers in women with or without TB infection, we analyzed cytokine levels in unstimulated whole blood obtained at different time points during pregnancy and postpartum (Fig. 2). In TB+ women, levels of three proinflammatory cytokines, IL-2, IP-10, and MCP-2, were higher postpartum than at 1st/2nd (IL-2: 5 pg/mL versus 2 pg/mL, *P* = 0.0021; IP-10: 331 pg/mL versus 76 pg/mL, *P* = 0.0001; and MCP-2: 80 pg/mL versus 45 pg/mL, *P* = 0.0001) and 3rd trimester (IL-2: 5 pg/mL versus 2 pg/mL, *P* = 0.0261; IP-10: 331 pg/mL versus 138 pg/mL, *P* = 0.007; and MCP-2: 80 pg/mL versus 47 pg/mL, *P* = 0.0001). We also compared nonspecific cytokine responses in women with and without TB infection at each defined time point. The postpartum levels of IL-2 and IP-10 were higher in TB+ women than those in TB− women (IL-2: 5 pg/mL versus 2 pg/mL, *P* = 0.0021 and IP-10: 331 pg/mL versus 87 pg/mL; *P* = 0.0001, respectively). Furthermore, TB+ women also had higher IP-10 levels at 3rd trimester than TB− women (138 pg/mL versus 68 pg/mL; *P* = 0.0311) (Fig. 3). No significant differences in the concentration of these cytokines were observed between the three time points in TB- women (Fig. S4).

### Correlation between cytokine responses during pregnancy and postpartum.

**FIG 4:**
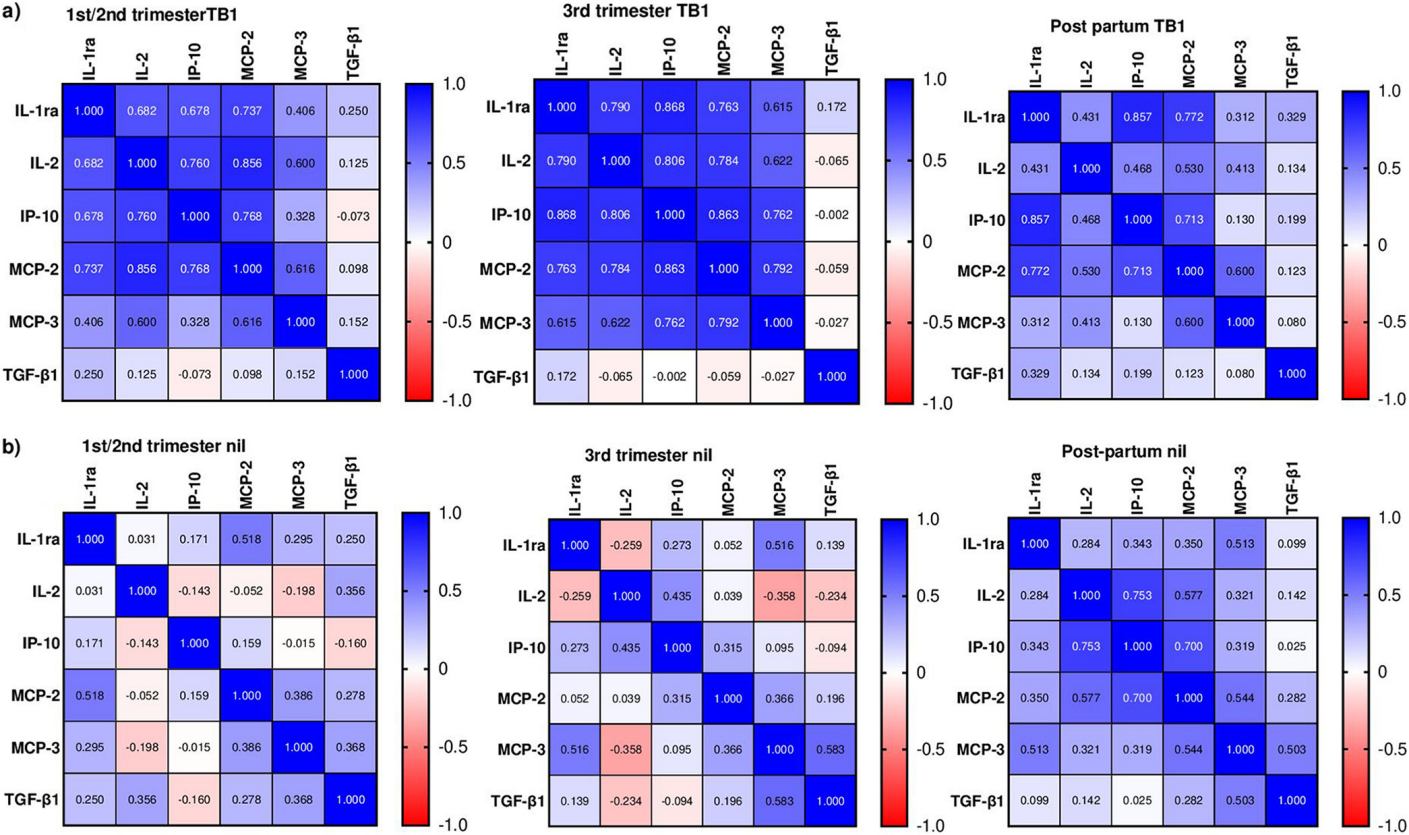
Correlation matrix for cytokine levels in: a) Mtb antigen (TB1)-stimulated and b) -unstimulated (nil) conditions at the 1st/2nd and 3rd trimesters and postpartum, including interleukin-1 receptor antagonist (IL-1ra); interleukin-2 (IL-2); IFN-γ inducible protein 10 (IP-10); Monocyte chemoattractant protein-2 (MCP-2); MCP-3; and transforming growth factor beta 1 (TGF-β1). Spearman Rank correlations were performed at each time point and r_s_-values are indicated along with color coding of correlation strength. Mtb, Mycobacterium tuberculosis.

To determine relationship between Mtb-specific cytokine responses at each time point, we performed correlation analyses. Mtb-triggered cytokine responses with a proinflammatory profile were positively correlated at all three time points (Fig. 4). The strongest correlations were found for TB1-triggered cytokines at 3rd trimester, which included: IP-10 versus IL-1ra, IL-2, MCP-2, and MCP-3 (r_s_ ≥ 0.76); IL-2 versus IL-1ra and MCP-2 (r_s_ ≥ 0.78); and MCP-2 versus IL-1ra and MCP-3 (r_s_ > 0.76) (for all correlations, *P* < 0.001). We also found strong positive correlations between levels of these cytokines at 1st/2nd trimester and postpartum (Fig. 4). Moreover, similar associations were found for TB2-stimulated cytokine levels (Fig. S5). However, no significant correlations were found between Mtb-antigen-stimulated secretion of TGF-β1 and the other cytokines. We also assessed correlations between cytokines in unstimulated samples and found significant correlation for IP-10 versus IL-2 and MCP-2 (r_s_ ≥ 0.70; *P* < 0.0001) postpartum (Fig. 4).

## DISCUSSION

In this longitudinal study, we explored the dynamics of Mtb-antigen cytokine secretion in women with and without TB infection, followed during pregnancy and postpartum. We observed significant alterations in the Mtb-stimulated cytokine profile, especially at later stages of pregnancy, with higher IL-2 and IP-10 expression and lower TGF-β1 secretion, compared to earlier stages of pregnancy. In addition, we noted that nonspecific IL-2, IP-10, and MCP-2 levels were elevated postpartum in TB+ women.

It is well established that pregnancy is accompanied by immune alterations occurring at the feto-maternal interface, as well as systemically (8, 20). These changes are more pronounced at later stages of pregnancy. Thus, pregnancy-driven hormonal changes shape the differentiation of CD4^+^ T-cell subsets and cytokines released from the corresponding cell types (20, 21). However, these immune modifications may also affect the immune control of latent infections., including TB.

The finding that Mtb-triggered IL-2 and IP-10 levels are elevated in late pregnancy is consistent with results of our previous study, which showed increased Mtb-antigen-stimulated IFN-γ secretion at the 3rd trimester in TB+ women, despite decreased mitogen-triggered responses (18). Taken together, these observations indicate elevated Mtb-antigen stimulation, during the pregnancy course, in TB+ women. It is possible that this is due to progression of TB infection, with increased bacterial activity in granulomas, inducing expansion of Mtb-specific T-cell populations in TB+ women. This phenomenon suggests that pregnancy affects the immune control of TB infection, with changes in the spectrum of TB infection (22), leading to incipient TB. However, since no cases of active TB were detected among our participants (neither during pregnancy nor during the 24-months follow up period after delivery) the Mtb-specific cellular responses triggered during pregnancy appear to counteract further progression and transition into active TB disease progression.

These observations stand in contrast to those of other studies, which have found lower levels of Mtb-specific Th1-induced cytokines at later stages of pregnancy (16, 23). A recent cross-sectional study conducted in TB+ pregnant women in Ethiopia indicated suppressed Th1 cellular responses, and unchanged or increased secretion of Th2 cytokines in response to Mtb antigen stimulation (24). Moreover, a longitudinal study from India also reported decreased levels of IFN-γ and IL-2 at delivery (23). Importantly, both these studies were restricted to women with HIV (23, 24), whereas our study only included HIV-uninfected women. We consider it likely that the discordant findings of these studies are due to failure of women with HIV to mount sufficient Mtb-specific immune responses to maintain control of TB infection. This mechanism could also explain the disproportionately high rates of pregnancy-associated active TB in women with HIV (15). Even though relatively few studies on TB infection in HIV-uninfected women have been published, lower Mtb-specific cytokine responses during pregnancy or close to delivery have been reported (25, 26). These findings stand in contrast to the current study, and previous study performed by us (18), but may be explained by the differences in timing of samplings during pregnancy and postpartum between these studies. For examples, in an Indian study (26), the follow-up samples were obtained within 5 days after delivery, whereas all our 3rd trimester samples were obtained before delivery (median 32 weeks of gestation). In turn, this may suggest highly dynamic immunological changes taking place close to, before and after, the delivery.

The findings of the present study also indicate different dynamics of T-cell subsets in response to perturbation of TB infection control during pregnancy. Mtb-specific secretion of IL-2 was higher both at 3rd trimester and postpartum than at 1st/2nd trimester. This pattern is in contrast to that of IP-10, and of IFN-γ previously reported by us (18), for which levels peaked at 3rd trimester. IL-2, which is primarily produced by memory T-cells, plays a critical role in cellular immunity and granuloma formation during TB infection (27). Following Mtb-antigen exposure, IL-2 released by activated Th1 cells stimulates T-cell growth, and proliferation and differentiation of effector T-cells, including cytotoxic T-cells (28–30). Elevated IL-2 levels have been reported in both TB disease and in latent TB infection (31, 32). Interestingly, Mtb-specific T-cells expressing IL-2 are predominantly found in individuals after completion of treatment for active TB (33). Thus, persistently elevated IL-2 levels, as noted postpartum in the present study, implies the expansion of Mtb-specific memory T-cells.

The secretion pattern of MCP-2 was similar to the pattern noted for IP-10. Mtb-specific expression of MCP-2 has also been observed in active TB, and its use as a biomarker for identification of active TB has been suggested (28). However, the exact role of MCP-2 during pregnancy remains to be elucidated. Still, upregulation of MCP-2 has been reported during preterm delivery and inflammation (34). The dynamic patterns of Mtb-specific cytokine alterations found during pregnancy are further supported by the relationships between Mtb-antigen–triggered release of IL-2 versus IP-10, MCP-2, and IL-1ra, with stronger correlations at 3rd trimester and weaker correlations postpartum. This finding is in line with increased Mtb-specific IP-10 and IL-2 responses previously reported in both active and latent TB (35). Furthermore, the combination of these cytokines improved the diagnostic performance of QFT IFN-γ in detecting Mtb infection (35). However, further validation studies are needed to determine the immunodiagnostic as well as the predictive value for TB progression in high-risk groups before these markers can be considered for clinical use.

Further, based on the upregulation of Treg-cells during pregnancy (9) and the role of Treg-cells in the pathogenesis of active TB (5, 36) we studied the dynamics of Mtb-specific TGF-β1 expression. The immunomodulating activities of Treg-cells involve production of regulatory cytokines, such as TGF-β1 (37, 38). TGF-β1 promotes fetal survival during pregnancy (39) and suppresses activity of macrophages and T-cells during Mtb infection (40). In the present study, we found lower Mtb-induced TGF-β1 levels at 3rd trimester than at earlier stages of pregnancy. Furthermore, these levels did not correlate with the levels of other cytokines, suggesting that the dynamics of Mtb-specific Treg-cells differ from those of T-cells expressing proinflammatory cytokines. A possible explanation could be that Mtb-specific memory Treg-cells migrate to the pulmonary sites of TB infection (41), similar to what has been demonstrated in experimental influenza pneumonitis (42).

Furthermore, we compared the longitudinal patterns of nonspecific cytokine secretion between women with and without TB infection. We found that levels of several cytokines, especially IL-2, IP-10, and MCP-2, were higher postpartum compared to during pregnancy in TB+ women. This result is consistent with lower levels of systemic proinflammatory cytokines during pregnancy in TB+ women, previously reported by Naik et al., (43). Interestingly, we observed higher levels of IP-10 in TB+ women than in women without TB infection, both at 3rd trimester and postpartum; a similar pattern was found for IL-2 levels postpartum.

Altogether, the current study reveals longitudinal expression profiles of Mtb-triggered cytokines, reflecting dynamic cellular immune responses during pregnancy in HIV-uninfected women with TB infection. Women with HIV are of special concern with regard to TB and pregnancy (23, 24), but knowledge on the immune balance in healthy women with TB infection is necessary to understand how pregnancy *per se* affects control of TB infection. The long follow-up of participants allowed us to exclude development of active TB despite immunological signs of elevated endogenous Mtb activity during pregnancy.

The inclusion of HIV-uninfected women was a strength of the study. However, inclusion of women with HIV from the same cohort, for comparison, could have further clarified the differences in immune control of TB infection in relation to HIV coinfection. Of the 1,834 women in our cohort, a minor proportion (9.3%) had HIV infection, and longitudinal series of samples were available from only a few of these. For this reason, and since the group of women with HIV was heterogeneous, with regard to degree of immunosuppression and antiretroviral treatment, we decided to limit the study to HIV-uninfected women.

Certain limitations of our study should be acknowledged. This includes the assessment of the dynamic changes of cytokine responses during pregnancy and postpartum in a relatively small sample of women. Other infections, such as cytomegalovirus, hepatitis E virus, herpes simplex virus, parasitic and helminth infections, could affect the Mtb nonspecific responses (44). However, these infections would not affect Mtb-specific cytokine responses, investigation of which was the main aim of this study. Other potential factors, such as alcohol use and smoking, that might influence the immune response, were rarely reported in our population.

In summary, we identified dynamic longitudinal changes in the secretion of Mtb-specific cytokines in women with TB infection, followed during pregnancy and postpartum, with elevated levels of IL-2 and IP-10, and reduced levels of TGF-β1, at the later stage of pregnancy. Women with TB infection also displayed higher levels of nonspecific IL-2 and IP-10 postpartum than women without TB infection. The Mtb-specific cytokine expression patterns indicate increased Mtb-antigen stimulation, suggesting progression of TB infection with enhanced Mtb activity during pregnancy. However, as none of these women developed active TB, we conclude that this phenomenon appears to be transient, and is counteracted by Mtb-specific immune responses in HIV-uninfected women.

## MATERIALS AND METHODS

### Participants and study procedure.

Participants for this study were identified from an ongoing prospective cohort study in Adama, Ethiopia, which was initiated for investigating different aspects of TB in relation to pregnancy (ClinicalTrials.gov identifier: NCT03305991) (45). Demographic and clinical data were collected at all visits, including bacteriological sputum investigations in women with symptoms suggestive of active TB (using smear microscopy, GeneXpert MTB/RIF PCR, and liquid culture). HIV testing was performed according to Ethiopian National guidelines. Nutritional status was assessed by measuring mid-upper arm circumference (MUAC).

At each study visit, blood samples were collected for TB infection testing using the QuantiFERON-TB Gold-Plus (QFT) assay according to the manufacturer’s instructions (Qiagen, Carnegie, Australia). Briefly, 1 mL of heparinized venous blood was dispensed into each QFT incubation tube (TB1- and TB2-antigens, negative control [nil], and positive control [mitogen]) within 8 h of venipuncture, and thereafter incubated at 37°C for 18 h. QFT Supernatants were collected after centrifugation and stored at −20°C for QFT ELISA. The remaining aliquots were stored at −80°C until further analysis, including shipment with maintained cold-chain.

We included all HIV-uninfected women with TB infection (TB+) from whom stored Mtb-stimulated whole blood supernatants collected at three time points: early pregnancy (1st or 2nd trimester), late pregnancy (3rd trimester), and 9 months after delivery (postpartum) were available. A control group of HIV-uninfected women without TB infection (TB−), sampled at the same three time points, was also included. As stated in recent World Health Organization consolidated guidelines on TB, we used the term “TB infection” instead of the previous terminology “latent TB infection” (46, 47). In this study, we defined TB infection as QFT IFN-γ concentration ≥0.35 IU/mL for TB1 and/or TB2 at all three time points, in the absence of active TB disease at inclusion and/or history of previous active TB. Absence of TB infection was defined as QFT IFN-γ concentration <0.2 IU/mL for TB1 and TB2 at all three time points.

### Analysis of immune mediators in QFT supernatants.

The concentration of immune mediators in Mtb-antigen-stimulated (TB1 and TB2) and -unstimulated (nil) whole blood supernatants was measured using Magnetic Luminex assay (R&D Systems, Minneapolis, MN) on the Bio-Plex 200 platform (Bio-Rad, Hercules, CA).

We initially, in a pilot study, measured levels of 32 immune mediators previously associated with pregnancy and TB (28, 48–51), including eotaxin, granzyme A, granzyme B, GM-CSF, IFN-γ, interferon-γ-induced protein 10 (IP-10), interleukins (IL-1β, IL-2, IL-4, IL-5, IL-6, IL-8, IL-10, IL-12 p70, IL-13, IL-15, IL-17a, and IL-21), IL-1 receptor antagonist (IL-1ra), MIG, *MIP*-1α, MIP-1β, monocyte chemoattractant protein-1, -2, and -3 (MCP-1, 2 and 3), osteopontin, PDGF-BB, RANTES, resistin, transforming growth factor β1 (TGF-β1), TNF-α, and VEGF. In this pilot study, the levels of 18 immune mediators were below the assay detection limits, and eight displayed no Mtb-specific response. Based on these results, six cytokines (IL-1ra, IL-2, IP-10, MCP-2, MCP-3, and TGF-β1) were included in the current analyses. The multiplex assay setup was designed with different dilutions as follows: HS IL-2 assay (1:3), TGF-β1 assay (1:15), MCP-2 and MCP-3 assay (1:3), and IP-10 and IL-1ra assay (1:30). Follow-up QFT supernatants, from the same individual, were analyzed on the same multiplex plate. The Mtb-antigen-specific cytokine responses were determined by subtracting the concentration detected in the nil supernatant from those detected in TB1 and TB2 supernatants. The cytokine concentrations below or above the assay detection limits were assessed by the lower or upper standard concentrations, respectively.

### Statistical analysis.

Longitudinal comparisons (three time points) were performed using Friedman-test, followed by Dunn’s multiple-comparison test. Cross-sectional comparisons between women with and without TB infection were performed using Mann-Whitney U-test and corrected for multiple comparisons using Bonferroni–Dunn method. Spearman Rank correlations were used to determine associations between cytokine responses at each study visit. Data were analyzed using GraphPad prism software version 9.0. *P*-value <0.05 was considered statistically significant.

### Ethical consideration.

This study was reviewed and approved by the National Research Ethics Review Committee, Addis Ababa, Ethiopia, and the Regional Ethical Review Board, Lund University, Sweden. Participants provided written informed consent to participate prior to enrollment.

### Data availability.

All experimental raw data on detected immune mediators are available upon request.
