# Supplementary material for: Dynamics of Mycobacterium tuberculosis-Specific and Nonspecific Immune Responses in Women with Tuberculosis Infection during Pregnancy
Source: Microbiol Spectr. 2022 Aug 15;10(5):e01178-22. doi: 10.1128/spectrum.01178-22 (PMC9603000; doi:10.1128/spectrum.01178-22)
Supplement: Supplemental file 1 — Supplemental material. Download spectrum.01178-22-s0001.pdf, PDF file, 0.5 MB [file spectrum.01178-22-s0001.pdf]

Supporting information

Supplementary Table 1. Concentrations of cytokines in unstimulated (nil) and Mtb-antigen (including TB1 and TB2) -stimulated whole blood supernatants from women longitudinally sampled during pregnancy and postpartum

Cytokine[a]	1st/2nd trimester	3rd trimester	Postpartum
**IL-1ra**			
nil	4945 (3569–8575)	3855 (2778–9142)	5345 (2537–8432)
TB1	6583 (3374–11741)	9528 (3191–26165)	10597 (5711–17336)
TB2	8415 (3215–16687)	11 377 (4788–25060)	7839 (3299–17296)
**IL-2**			
nil	2 (2–2)	2 (2–2)	5 (2–14)
TB1	62 (44–120)	139 (57–346)	175 (76–234)
TB2	79 (50–122)	138 (61–397)	159 (66–229)
**IP-10**			
nil	76 (62–127)	138 (69–260)	331 (181–590)
TB1	2310 (1111–4125)	4999 (1743–7280)	4186 (1341–6927)
TB2	3026 (1033–5860)	5865 (2350–7374)	3729 (1517–6820)
**MCP-2**			
nil	45 (33–56)	47 (29–57)	80 (57–182)
TB1	321 (198–1536)	658 (249–1982)	1239 (564–1982)
TB2	1026 (211–1800)	714 (296–2132)	1268 (553–2055)
**MCP-3**			
nil	1751 (942–3751)	1202 (440–2944)	1164 (910–2977)
TB1	1308 (-1 to 2522)	471 (-149 to 1504)	2229 (939–5708)
TB2	1831 (275–4598)	382 (-55 to 1807)	2213 (698–5582)
**TGF-β1**			
nil	67.3 (51.1–93.3)	76.8 (63.1–92.5)	79.9 (66.3–103.1)
TB1	2.3 (-7.9 to 21.2)	-6.8 (-13.6 to -0.3)	0.5 (-26.5 to 9.1)
TB2	-1.7 (-6.7 to 10.9)	-7.1 (-15.0 to 1.6)	-0.9 (-16.0 to 29.0)

[a]Concentrations (pg/mL for all, except ng/mL for TGF-β1) (median and interquartile range in parentheses) of each cytokine elicited in supernatants of unstimulated (nil) and Mtb-antigen (TB1 and TB2) stimulated (after subtracting nil concentrations) whole blood. IL-1ra, interleukin-1 receptor antagonist; IL-2, interleukin 2; IP-10, IFN-γ inducible protein 10; MCP-2; MCP -3, Monocyte chemoattractant protein; TGF-β1, Transforming growth factor beta 1; Mtb, *Mycobacterium tuberculosis*.

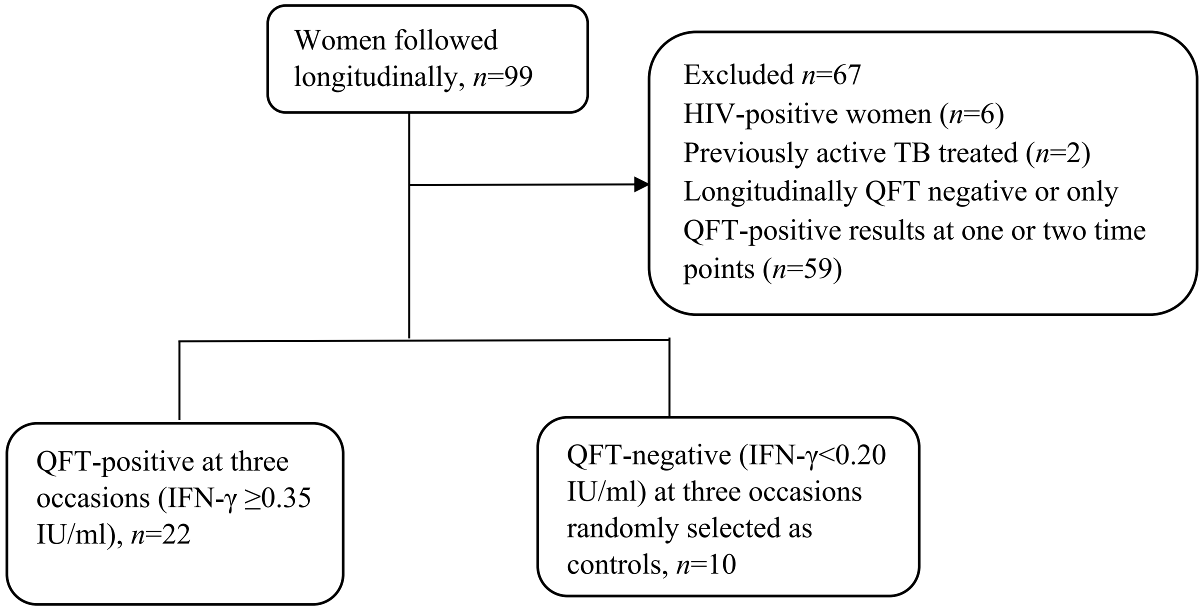

Supplementary Figure 1. Flow chart of pregnant women followed during pregnancy and postpartum, included for analysis of longitudinal Mtb-stimulated (TB1 and TB2) and -unstimulated (nil) cytokines (IL-1ra, IL-2, IP-10, MCP-2, MCP-3, and TGF-β1) responses. IL-1ra, interleukin-1 receptor antagonist; IL-2, interleukin 2; IFN, interferon; IP-10, IFN-γ inducible protein 10; MCP, Monocyte chemoattractant protein; TGF-β1, Transforming growth factor beta 1; Mtb, *Mycobacterium tuberculosis*

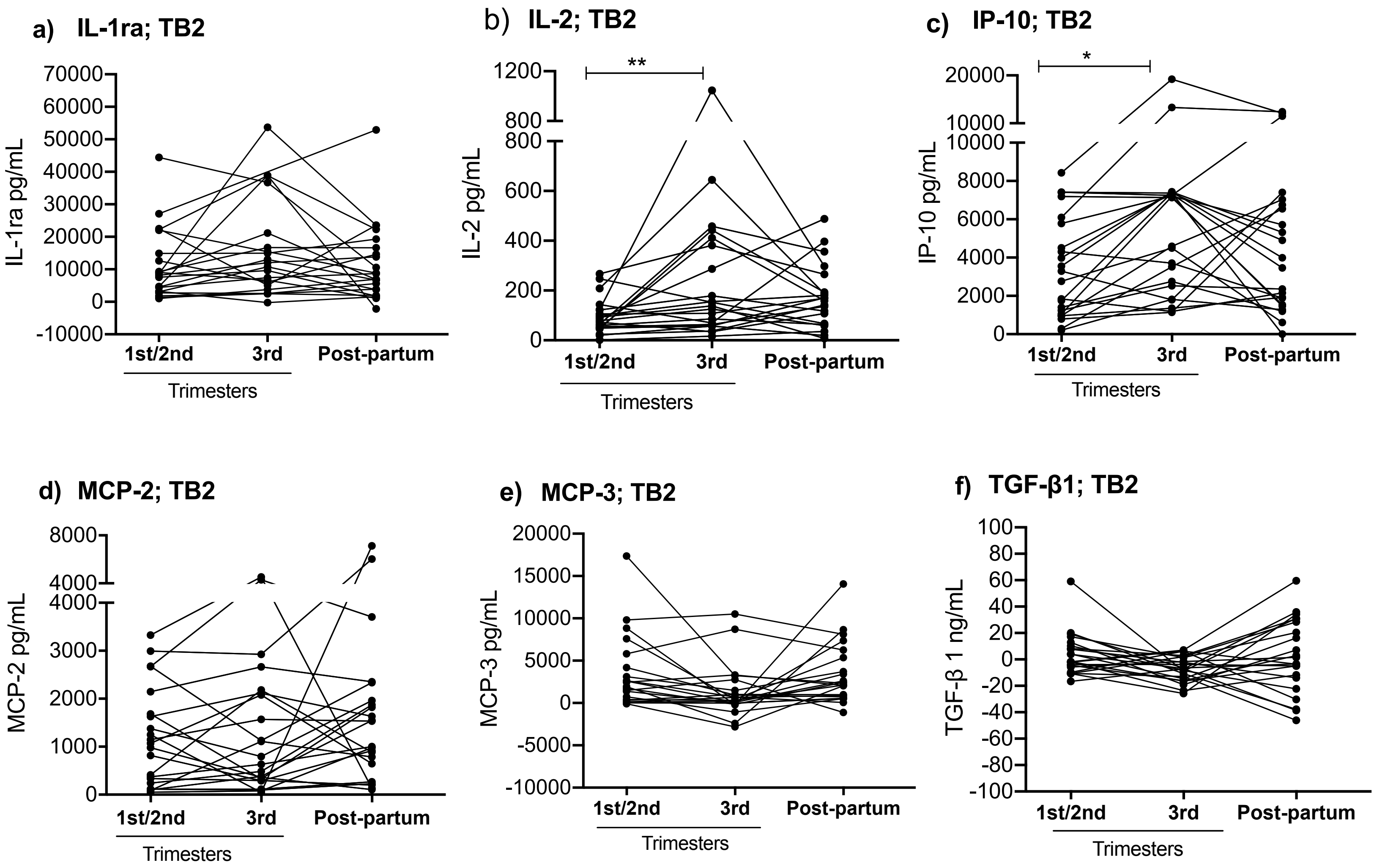

a) IL-1ra; TB2
IL-1ra pg/mL
1st/2nd
3rd
Post-partum
Trimesters
b) IL-2; TB2
IL-2 pg/mL
**
c) IP-10; TB2
IP-10 pg/mL
*
d) MCP-2; TB2
MCP-2 pg/mL
e) MCP-3; TB2
MCP-3 pg/mL
f) TGF-β1; TB2
TGF-β 1 ng/mL

Supplementary Figure 2. Mtb-antigen (TB2)-stimulated cytokine responses (IL-1ra, IL-2, IP-10, MCP-2, MCP-3, and TGF-β1) in women longitudinally tested at the $1^{st}/2^{nd}$ and 3rd trimesters and postpartum ($n = 22$). Longitudinal comparisons were performed using Friedman test, followed by Dunn's multiple comparisons. *$p < 0.05$ and **$p < 0.01$. IL-1ra, interleukin 1 receptor antagonist; IL-2, interleukin-2; IP-10, IFN-γ inducible protein 10; MCP-2, Monocyte chemoattractant protein-2; MCP-3; TGF-β1, Transforming growth factor beta 1; Mtb, *Mycobacterium tuberculosis*.

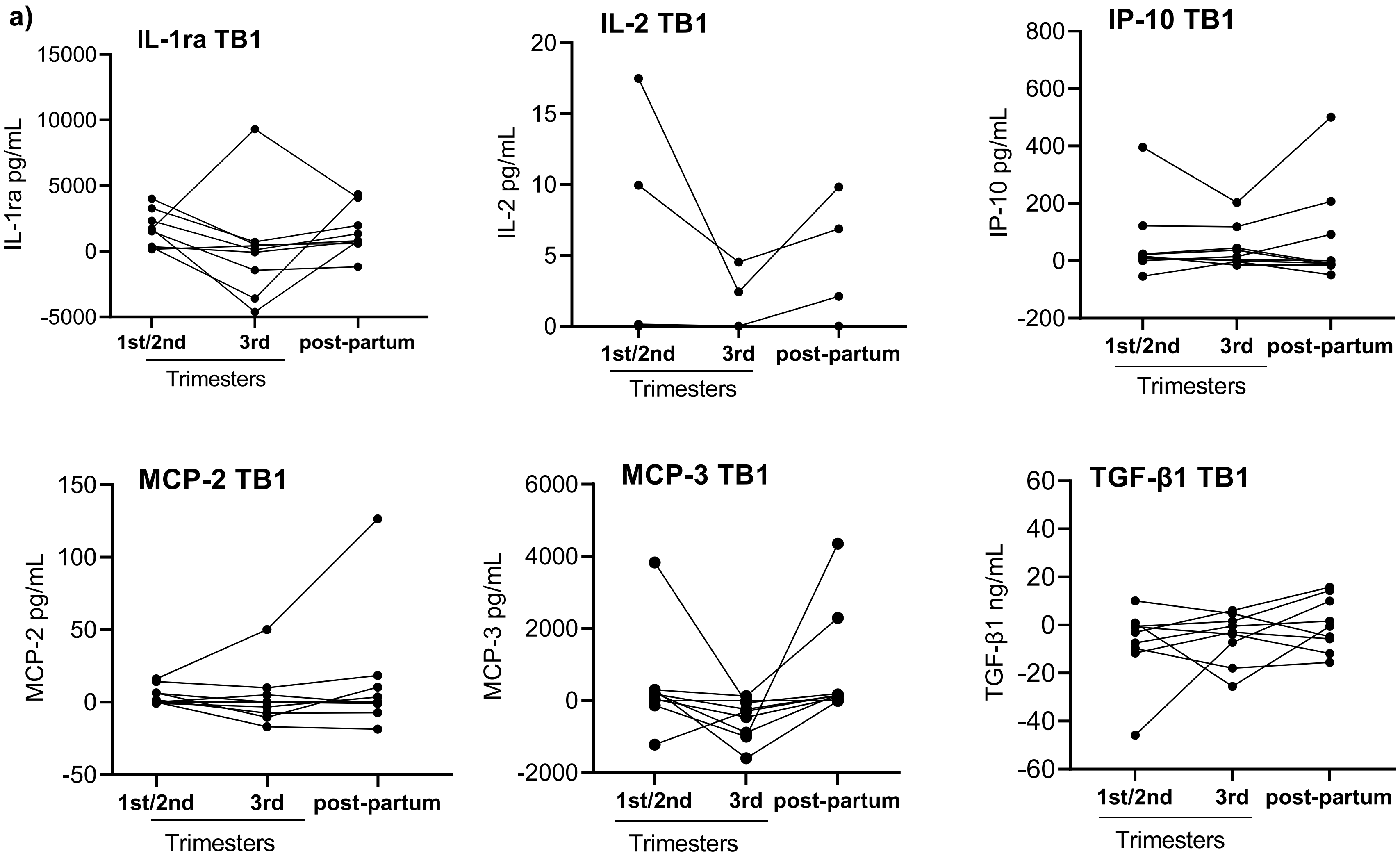

a)
IL-1ra TB1
IL-1ra pg/mL
15000
10000
5000
0
-5000
1st/2nd
3rd
post-partum
Trimesters
IL-2 TB1
IL-2 pg/mL
20
15
10
5
0
1st/2nd
3rd
post-partum
Trimesters
IP-10 TB1
IP-10 pg/mL
800
600
400
200
0
-200
1st/2nd
3rd
post-partum
Trimesters
MCP-2 TB1
MCP-2 pg/mL
150
100
50
0
-50
1st/2nd
3rd
post-partum
Trimesters
MCP-3 TB1
MCP-3 pg/mL
6000
4000
2000
0
-2000
1st/2nd
3rd
post-partum
Trimesters
TGF-β1 TB1
TGF-β1 ng/mL
60
40
20
0
-20
-40
-60
1st/2nd
3rd
post-partum
Trimesters

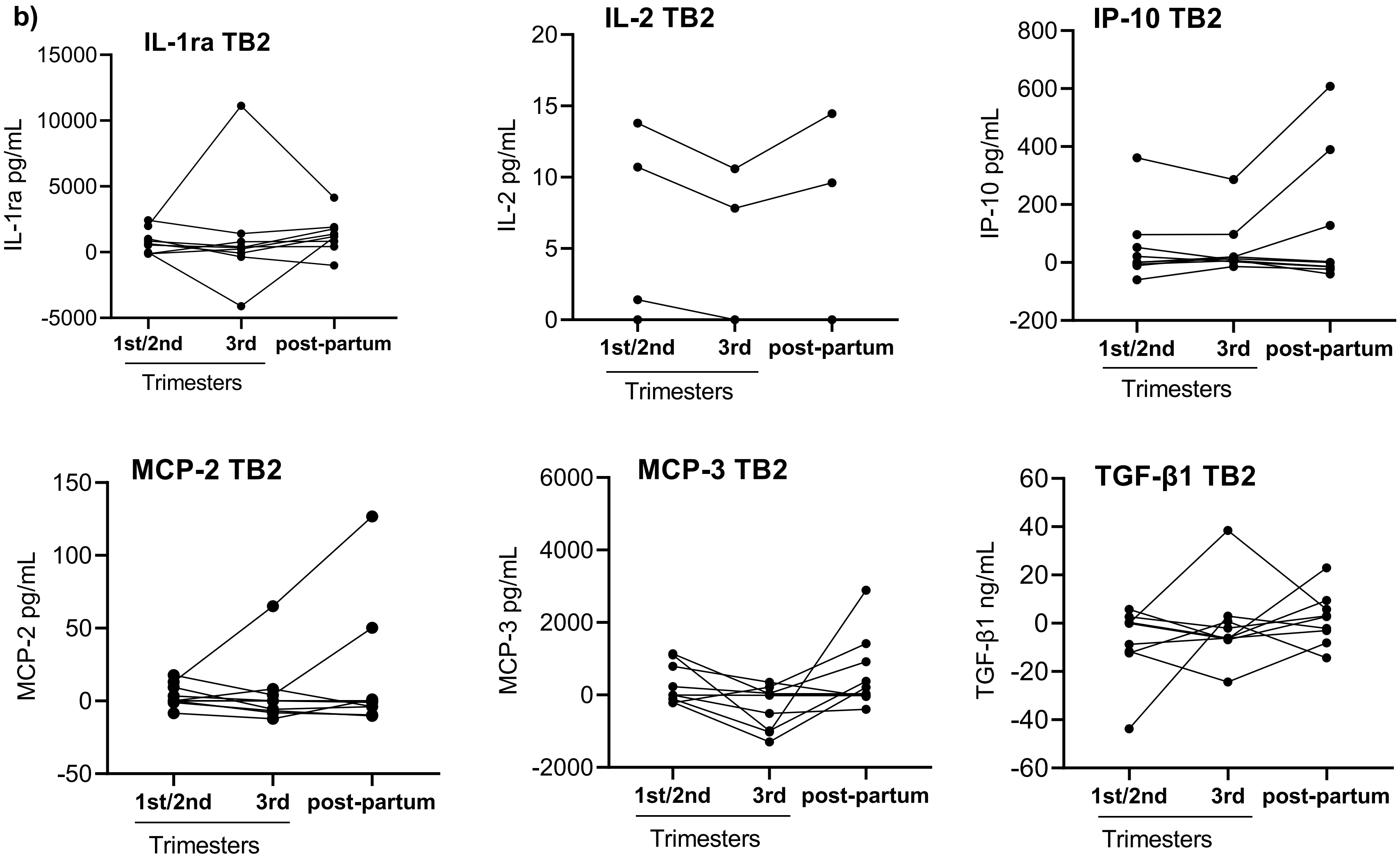
b)
IL-1ra TB2
IL-1ra pg/mL
15000
10000
5000
0
-5000
1st/2nd
3rd
post-partum
Trimesters
IL-2 TB2
IL-2 pg/mL
20
15
10
5
0
1st/2nd
3rd
post-partum
Trimesters
IP-10 TB2
IP-10 pg/mL
800
600
400
200
0
-200
1st/2nd
3rd
post-partum
Trimesters
MCP-2 TB2
MCP-2 pg/mL
150
100
50
0
-50
1st/2nd
3rd
post-partum
Trimesters
MCP-3 TB2
MCP-3 pg/mL
6000
4000
2000
0
-2000
1st/2nd
3rd
post-partum
Trimesters
TGF-β1 TB2
TGF-β1 ng/mL
60
40
20
0
-20
-40
-60
1st/2nd
3rd
post-partum
Trimesters

Supplementary Figure 3. Mtb-antigen stimulated cytokine responses in a) TB1 and b) TB2, for IL-1ra, IL-2, IP-10, MCP-2, MCP-3, and TGF-β1 in TB- women (controls) longitudinally tested at the $1^{st}/2^{nd}$ and 3rd trimesters and postpartum (n = 10). Ten women were also tested for IL-2 responses, however, since several data points ended up at 0, these are not visualized in the graphs. Longitudinal comparisons were performed using Friedman test, followed by Dunn's multiple comparison test. *$p < 0.05$ and **$p < 0.01$. IL-1ra, interleukin 1 receptor antagonist; IL-2, interleukin-2; IP-10, IFN-γ inducible protein 10; MCP-2, Monocyte chemoattractant protein-2; MCP-3, Monocyte chemoattractant protein-3; TGF-β1, Transforming growth factor beta 1; Mtb, *Mycobacterium tuberculosis*.

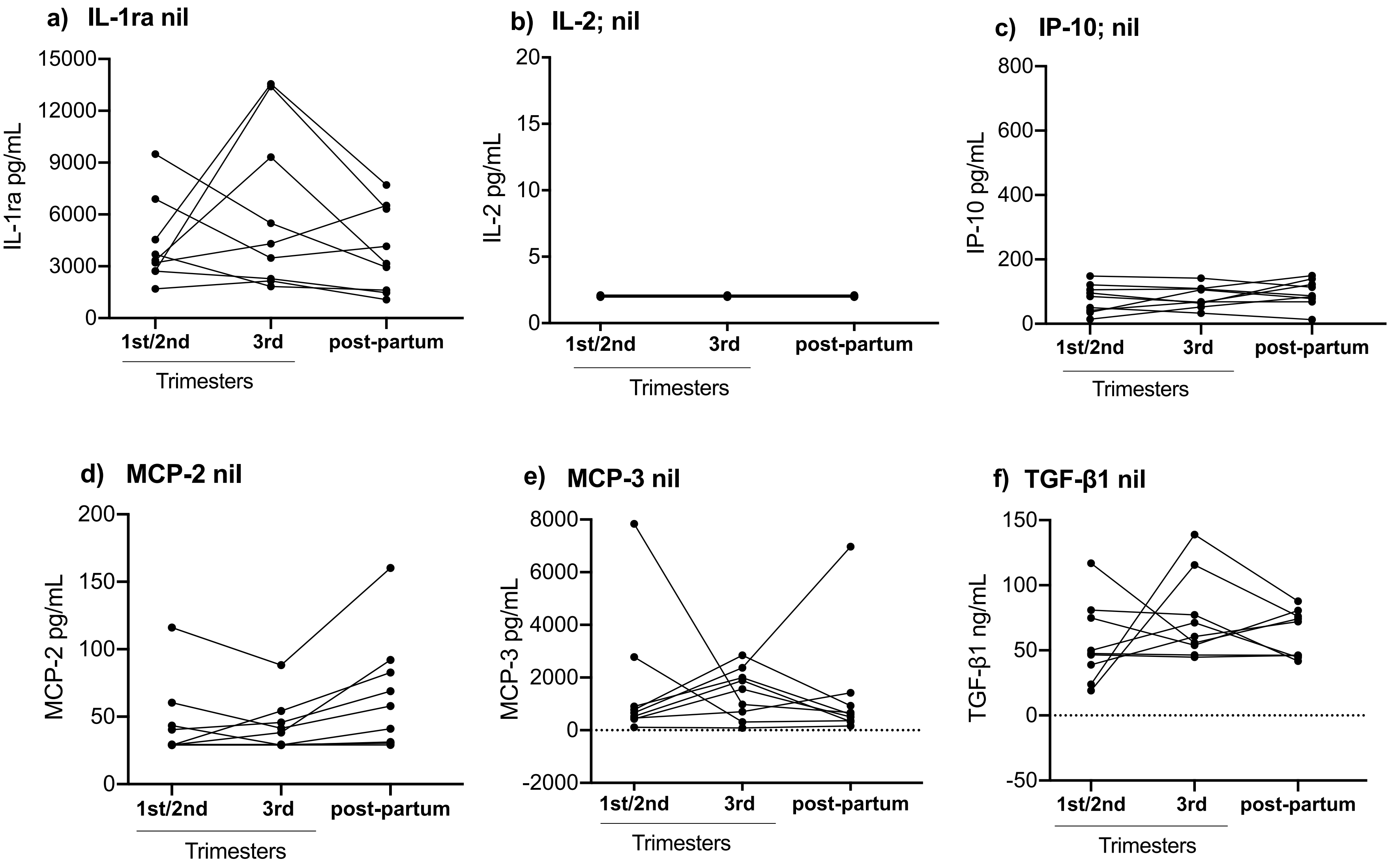
a) IL-1ra nil
15000
12000
9000
6000
3000
0
IL-1ra pg/mL
1st/2nd
3rd
post-partum
Trimesters
b) IL-2; nil
20
15
10
5
0
IL-2 pg/mL
1st/2nd
3rd
post-partum
Trimesters
c) IP-10; nil
800
600
400
200
0
IP-10 pg/mL
1st/2nd
3rd
post-partum
Trimesters
d) MCP-2 nil
200
150
100
50
0
MCP-2 pg/mL
1st/2nd
3rd
post-partum
Trimesters
e) MCP-3 nil
8000
6000
4000
2000
0
-2000
MCP-3 pg/mL
1st/2nd
3rd
post-partum
Trimesters
f) TGF-β1 nil
150
100
50
0
-50
TGF-β1 ng/mL
1st/2nd
3rd
post-partum
Trimesters

Supplementary Figure 4. Concentrations of IL-1ra, IL-2, IP-10, MCP-2, MCP-3, and TGF-β1 in unstimulated whole blood supernatants longitudinally analysed at the 1st/2nd and 3rd trimesters and postpartum in TB- women (QFT <0.20 IU/mL, *n* = 10). Ten women were also tested for IL-2 responses, however, since several data points ended up at 2, these are not visualized in the graph. Longitudinal comparisons were performed using Friedman test, followed by Dunn's multiple comparisons. IL-1ra, interleukin 1receptor antagonist; IL-2, interleukin-2; IP-10, IFN-γ inducible protein 10; MCP-2, Monocyte chemoattractant protein-2; MCP-3, Monocyte chemoattractant protein-3; TGF-β1, Transforming growth factor beta 1; QFT, QuantiFERON TB Gold Plus.

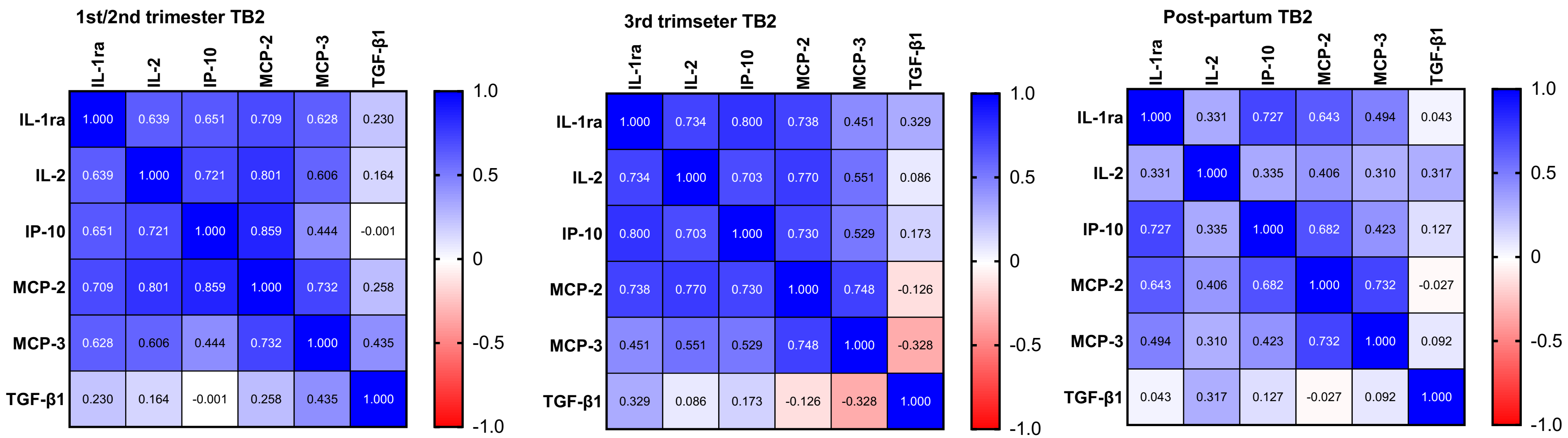

Supplementary Figure 5. Correlations between cytokines (IL-1ra, IL-2, IP-10, MCP-2, MCP-3, and TGF-β1) in TB antigen (TB2) stimulated supernatants at the 1st/2nd and 3rd trimesters and postpartum. Spearman's correlations were performed at each time point.
